# Macro-reentrant Single-loop Biatrial Flutter Appearing as Typical Atrial Flutter: Case Study and Review

**DOI:** 10.19102/icrm.2020.111106

**Published:** 2020-11-15

**Authors:** Aneesh V. Tolat, Elizabeth Clark, Vamsi Naraparaju, Joseph E. Flack

**Affiliations:** ^1^Electrophysiology Section, Hoffman Heart and Vascular Institute, St. Francis Hospital and Medical Center, University of Connecticut School of Medicine, Hartford, CT, USA; ^2^Department of Cardiothoracic Surgery, Hoffman Heart and Vascular Institute, St. Francis Hospital and Medical Center, University of Connecticut School of Medicine, Hartford, CT, USA

**Keywords:** Ablation, atrial flutter, atypical flutter, Bachmann’s bundle, cardiac surgery, coronary sinus

## Abstract

Biatrial flutter is a rare form of macro-reentrant atrial tachycardia that involves both the right and left atria. Single-loop biatrial flutter is typically associated with scarring of the septum from prior ablation or surgery and is generally made up of two interatrial connections—that is, the coronary sinus and Bachmann’s bundle. Entrainment and high-density mapping allow for rapid diagnosis and development of a treatment strategy. Ablation planning should also take into consideration the preservation of interatrial conduction. We herein discuss a case of single-loop biatrial flutter presenting as a typical atrial flutter and review the differential diagnosis and physiology of the arrhythmia.

## Introduction

Typical cavotricuspid isthmus (CTI)-dependent atrial flutter has a distinct, easily recognized pattern on the electrocardiogram (ECG) and has become a readily ablated single-loop reentrant arrhythmia in the right atrium (RA). Occasionally, during electrophysiology (EP) study, other patterns of arrhythmia are found that mimic typical atrial flutter, especially in patients with preexisting scar tissue from prior surgery, ablation, or inflammatory disease. Biatrial loop tachycardia, a rare form of reentrant arrhythmia associated with scars on the septum, can be more challenging to treat. As such, a thorough understanding of its possible mechanisms is necessary before successful ablation can be performed. We present the case of a patient with typical-appearing atrial flutter in whom a single-loop tachycardia with activation around the tricuspid and mitral valve was found and discuss the differential diagnosis as well as the substrate requirements of the tachycardia and considerations for ablation.

## Case presentation

**Figure 1:**
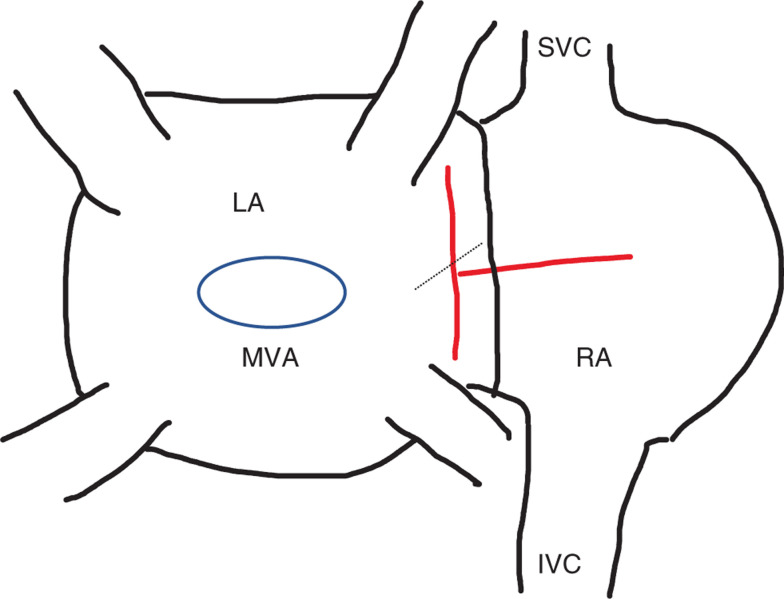
Diagram of surgical incisions from the posterior view performed during mitral valve surgery. An interatrial groove vertical incision was made between the right-sided pulmonary veins and septum in the LA (shown in red) with extension of the incision to the outer wall of the RA (also shown in red). The septum on the inside of the RA was then incised and opened (blue dashed line) toward the mitral valve annulus. IVC: inferior vena cava; LA: left atrium; MVA: mitral valve annulus; RA: right atrium; SVC: superior vena cava.

**Figure 2:**
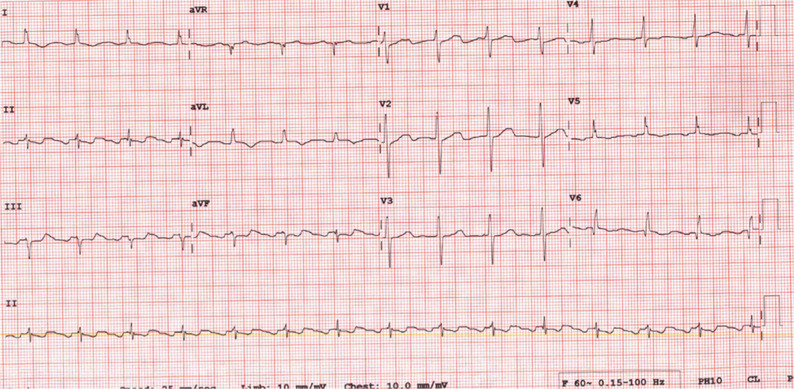
Twelve-lead ECG of the atrial flutter.

A 56-year-old man with diabetes, hypertension, and coronary disease presented with severe mitral regurgitation and underwent mitral valve replacement with coronary artery bypass grafting (CABG) in April 2019. The surgeon initially performed a vertical incision in the left atrium (LA) between the right pulmonary veins and the interatrial septum in the interatrial groove (Waterston’s groove). However, because of poor visualization, another horizontal incision was made in the posterior RA and included the atrial septum **(Figure 1)**. Thereafter, mitral valve replacement was performed through this larger incision involving the RA, septum, and LA. Postoperatively, the patient developed atrial fibrillation and was placed on amiodarone for several weeks and then taken off the medication. Several months later, he developed an atrial flutter as shown in **Figure 2**. The ECG showed a slow atrial flutter with morphology consistent with that of a typical atrial flutter.

The patient was placed on anticoagulation and, after discussion, wished to proceed with a cardiac EP study. The patient was brought to the EP laboratory and catheters were placed in the coronary sinus (CS) and anterolateral RA (proximal catheter in the high lateral RA, with distal electrodes inferior toward the floor of the RA along the low lateral wall), along with a His-bundle catheter (HBE).

Entrainment mapping was performed from the proximal CS (PCS) **(Figure 3A)**, low lateral RA (Deca 1–2) **(Figure 3B)**, and distal CS (DCS) **(Figure 3C)**. The postpacing interval (PPI) for each of the sites was within 20 ms of the tachycardia cycle length (TCL), suggesting a biatrial tachycardia as depicted in **Figure 3D**. The ablation catheter (Abl) was then placed on the CTI **(Figure 3E)**. Given that both adjacent sides (RA low lateral wall and PCS) of the CTI appeared to be within the circuit, ablation was conducted along the CTI, prolonging the CL from 340 ms to between 370 and 380 ms, but did not terminate the tachycardia.

As a result, repeat entrainment mapping was performed from the low lateral RA (Deca 1–2), PCS, and DCS with the longer CL atrial flutter and the PPIs were found to be within 30 ms of the TCL, again suggesting the involvement of both atria in the tachycardia.

**Figure 4:**
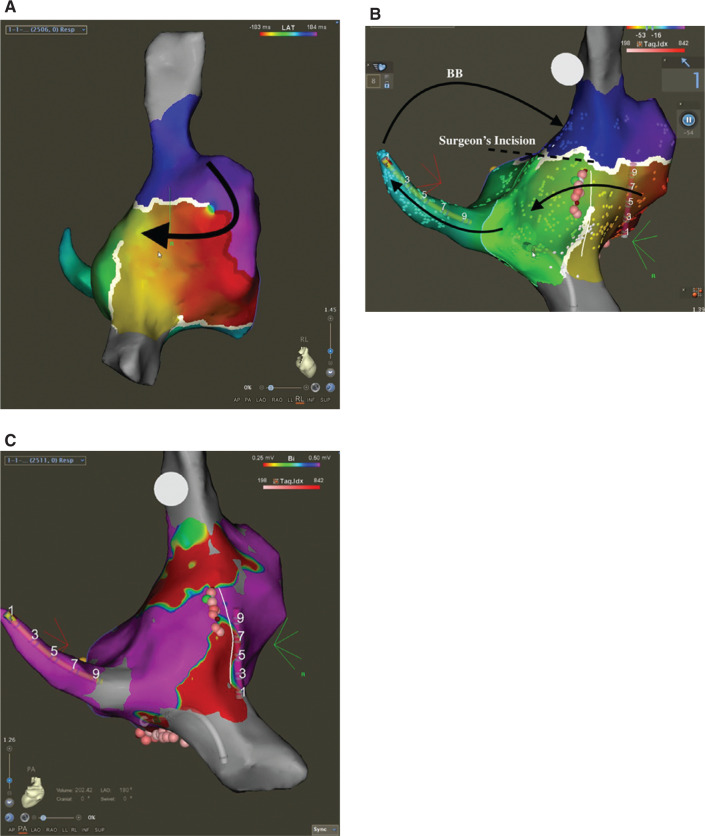
**A:** Color activation map of the right lateral RA after CTI ablation with prolongation of the CL. Activation was seen occurring down the lateral wall from BB and then moving posteriorly toward the CS. **B:** Activation map posterior view of the RA after RA isthmus ablation covering 360 ms out of the 380 ms of the TCL. A narrow channel is seen between the SVC and IVC facilitating conduction from the lateral wall to the CS. The map also shows that the light blue portion of the CL is missing, corresponding with activation of BB. The surgeon’s incision across the septum is also noted. **C:** Posterior view of the RA, with a voltage map of the RA showing scar and low-voltage areas in red forming a narrow channel, across which activation was found to occur. Catheters are seen in the CS (yellow) and posterior RA (red). Ablation lesions are seen across the channel, which terminated the tachycardia. BB: Bachmann’s bundle; CS: coronary sinus; RA: right atrium; TCL: tachycardia cycle length.

A multielectrode catheter (Pentaray; Biosense Webster, Diamond Bar, CA, USA) was used to perform high-density activation mapping in the RA and in the CS. **Figures 4A and 4B** show activation occurring from the anterior RA down the lateral wall of the RA, then moving to the posterior RA above the inferior vena cava (IVC). Subsequent activation of the septum then occurred, followed by proximal to distal CS activation. High-density voltage mapping **(Figure 4C)** was also used to delineate scar from prior surgery. When combined with high-density activation mapping, the electroanatomic map showed activation occurring through a channel on the posterior wall that was bordered by scar. It also revealed that a portion of the circuit was still missing (light blue color) between the DCS and anterior RA, consistent with activation over Bachmann’s bundle (BB). Mapping of this remaining portion of the circuit would have required access to the LA and was not deemed necessary and was not performed to avoid unnecessary procedural risk to the patient.

**Figure 5:**
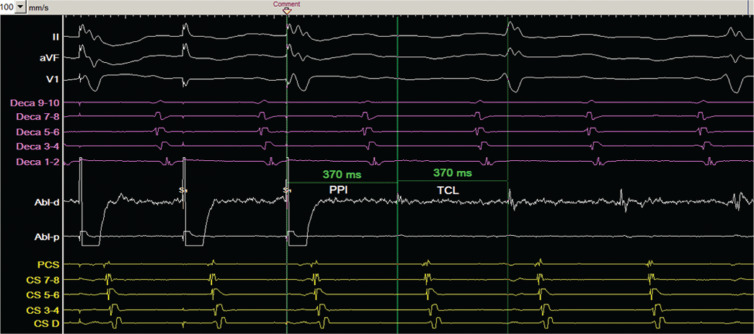
Entrainment from the distal ablation catheter near the narrow channel seen on the posterior RA. The PPI was 370 ms and was equal to the TCL. Intracardiac recordings include the anterolateral RA catheter (Deca) in purple, ablation catheter in white, and CS catheter in yellow. The distal electrodes of the anterolateral RA catheter are inferior, while the proximal electrodes are superior along the RA free wall. Abl: ablation catheter; CS: coronary sinus; RA: right atrium; TCL: tachycardia cycle length.

Entrainment was performed adjacent to the posterior RA channel **(Figure 5)**, with a PPI of 370 ms that was identical to the TCL. Pacing from the ablation catheter at a high output of 15 mA was also performed to exclude the possibility of ablation of the phrenic nerve. Ablation in this area slowed and terminated the tachycardia, which could not be induced at the end of the procedure.

**Figure 6:**
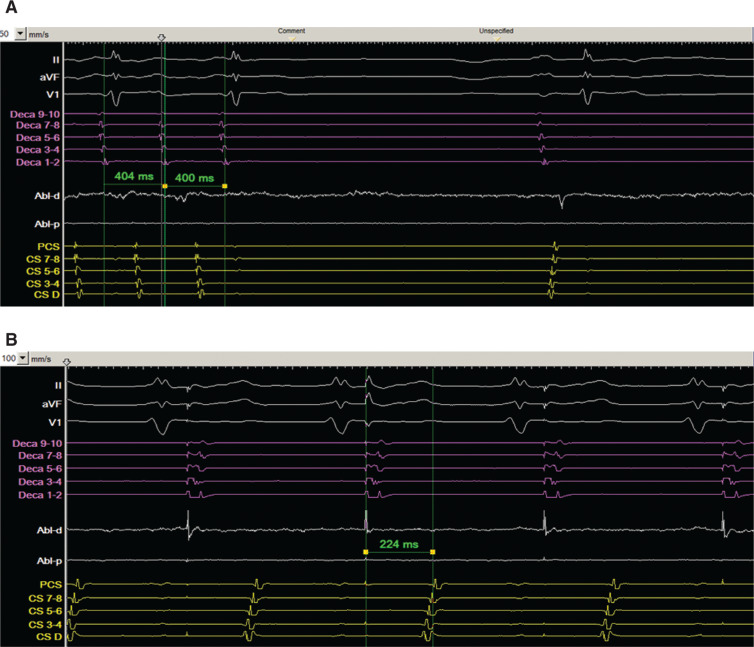
**A:** Ablation near the posterior RA channel terminated atrial flutter to sinus rhythm. Left atrial activation occurred over BB as the CS was isolated from the RA. **B:** Pacing from the low anterolateral RA to check the CTI ablation line. Conduction at this point was now occurring from distal to proximal within the CS, while time to the PCS had become markedly prolonged at 224 ms. In **A** and **B**, intracardiac recordings include the anterolateral RA catheter (Deca) in purple, Abl in white, and CS catheter in yellow. Also, the distal electrodes of the anterolateral RA catheter are inferior, while the proximal electrodes are superior along the RA free wall. BB: Bachman’s bundle; Abl: ablation catheter; PCS: proximal coronary sinus; RA: right atrium.

In addition, importantly, while attempting to check the line of block at the CTI by pacing the lateral portion of the CTI, activation of the LA was noted to change postablation **(Figures 6A and 6B)**, occurring over BB with DCS activation occurring prior to PCS as compared to while in tachycardia.

## Discussion

The ablation of typical RA flutter as recognized by the 12-lead ECG has become an effective treatment for symptomatic patients. However, not all atrial flutters that appear typical can be successfully treated by ablation at the CTI. In this case, entrainment mapping demonstrated findings consistent with atrial flutter involving the RA and LA, suggesting the role of both chambers in the condition.^1^

Ip et al. elegantly outlined the differential diagnosis of such a patient with entrainment from low lateral RA, PCS, and DCS with a PPI − TCL value within 30 ms.^2^ The differential diagnosis included (1) single-loop macro-reentry involving the RA and LA, (2) double-loop reentry in a figure-of-eight pattern around the tricuspid and mitral valves, and (3) RA flutter with LA participating as a passive bystander during entrainment. In this case, double-loop reentry around the tricuspid and mitral valves as first reported by Shah et al.^3^ was excluded by the direction of CS activation with the RA activated in a counterclockwise pattern. Here, activation occurred from the PCS to DCS, while figure-of-eight dual-loop reentry would be expected to occur from the DCS to PCS.

The possibility of RA flutter with the LA participating as bystander for the initial atrial flutter CL of 340 ms was not easy to exclude and, during PCS entrainment, we did not observe the His-atrial electrogram (His A) to follow (concept of linking between the LA and RA) the DCS activation (parallel versus serial activation) as mentioned in the paper by Ip et al.^2^ While this might suggest that the LA is a bystander to the RA, this phenomenon might also be explained by local His A conduction during PCS entrainment with slow conduction or block distal to the conducted His A.

After CTI ablation, which was associated with slowing of the tachycardia, the conduction time increased from the low lateral RA to the PCS. Repeat entrainment mapping following CTI isthmus ablation was consistent with continued involvement of both the RA and LA. High-density electroanatomic mapping was conducted after the CTI line and showed activation of the RA occurring posteriorly across a small channel of tissue formed by scarring from a previous surgery, which was used to create a line of block and terminate the arrhythmia.

High-density electroanatomic activation mapping played an important and complementary role in this case by helping to explain the response to entrainment mapping seen with the second atrial flutter. High-density mapping allowed for visualization of low-voltage areas from the patient’s prior surgery acting as anatomic barriers along with the activation pattern, which involved both the RA and LA.

Biatrial tachycardias, while a rare form of macro-reentrant tachycardia, are increasingly being recognized and are usually the result of either previous ablation, surgery, or intrinsic disease/scarring along the septum.^2–6^ Kitamura et al. used ultra-high-resolution mapping to describe three different types of biatrial tachycardia found in their series.^6^ Among 422 atrial tachycardias in their series, nine biatrial tachycardias (2.1%) were identified, of which three (33%) were classified as involving single-loop reentry around the mitral and tricuspid valves with two interatrial connections. The other two types of biatrial tachycardia categories described included a reentrant circuit encompassing most of the mitral annulus and RA septum with two interatrial connections and a reentrant circuit involving the LA septum and RA septum with two interatrial connections, respectively. These authors also noted that septal scar was an important requisite for biatrial tachycardia together with the presence of two interatrial connections (usually BB and the CS) to allow for circus movement tachycardia. In the present case, the surgical scar along the septum (second incision) with enlarged atria likely predisposed the patient to biatrial tachycardia.

Ablation at the CTI of the single-loop biatrial flutter around the tricuspid and mitral valves (using the CS and BB as interatrial connections) would normally be expected to terminate the arrhythmia. However, in our case, the atrial flutter changed and slowed, ultimately finding another way to reach the PCS via a posterior RA channel. Ultimately, by mapping and ablating the second RA posterior channel, the tachycardia was terminated to the sinus. This, however, also limited sinus activation to the LA via BB as evidenced by the activation of the DCS being earlier than that of the PCS.

Targeting the site of ablation in biatrial tachycardia usually involves the identification of a critical isthmus requisite for the tachycardia to continue. For those circuits involving the valves, ablation ideally should be performed at the RA or LA isthmus^6^; however, for other types of biatrial tachycardia, care should be taken to avoid complete disconnection of the interatrial bridging at the level of BB or the CS.

## Conclusions

Single-loop biatrial tachycardia around the mitral and tricuspid valves is a rare form of macro-reentrant tachycardia involving both the RA and LA that can be diagnosed by entrainment as well as activation mapping. A septal scar with the presence of two interatrial connections is required for the tachycardia to occur and ablation at the RA isthmus usually is successful in eliminating this tachycardia. In this case, termination did not occur with CTI ablation but, instead, changed to a second atrial flutter requiring a second ablation line for termination, which also eliminated RA to CS activation of the LA.

## Figures and Tables

**Figure 3:**
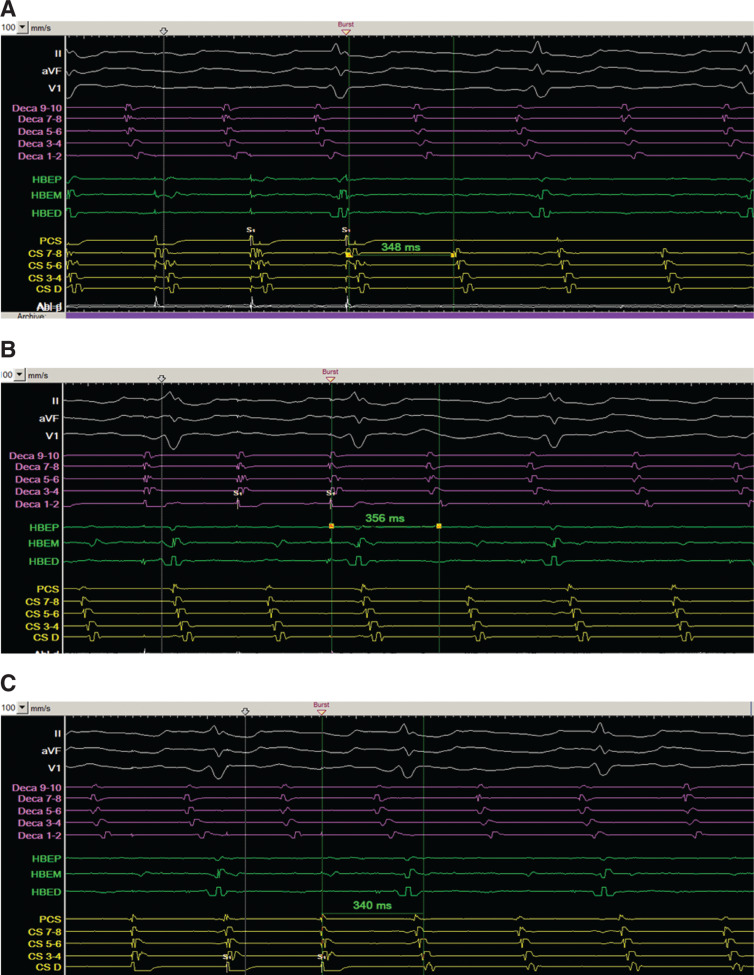
**A:** Entrainment from the CS with a PPI of 348 ms and TCL of 340 ms. **B:** Entrainment from the distal anterolateral RA with a PPI of 356 ms and TCL of 340 ms. **C:** Entrainment from the distal CS with a PPI of 340 ms and TCL of 340 ms. In **A**, **B**, and **C**, intracardiac recordings include the anterolateral RA catheter (Deca) in purple, Abl, and CS catheter in yellow. Also, the distal electrodes of the anterolateral RA catheter are inferior, while the proximal electrodes are superior along the RA free wall. CS: coronary sinus; HBE: His-bundle catheter; PCS: proximal coronary sinus.

**Figure 3:**
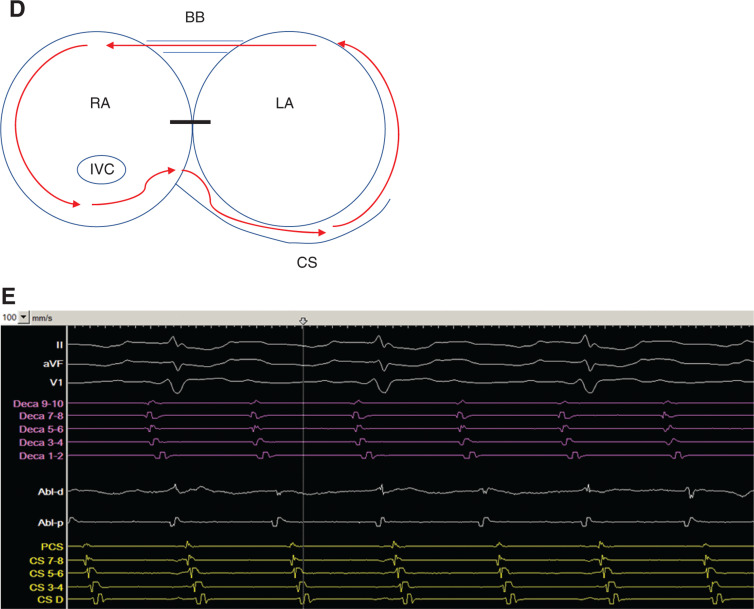
**D:** Diagram of proposed initial flutter circuit involving the RA and LA. The black bar indicates scar tissue from prior surgery. CS: coronary sinus. **E:** Abl positioned at the CTI prior to RF ablation. In **E**, intracardiac recordings include the anterolateral RA catheter (Deca) in purple, Abl, and CS catheter in yellow. Also, the distal electrodes of the anterolateral RA catheter are inferior, while the proximal electrodes are superior along the RA free wall. BB: Bachmann’s bundle; CS: coronary sinus; HBE: His-bundle catheter; IVC: inferior vena cava; LA: left atrium; PCS: proximal coronary sinus; RA: right atrium.
